# Supplementary material for: Stacking Model‐Based Classifiers for Dealing With Multiple Sets of Noisy Labels
Source: Biom J. 2025 Mar 12;67(2):e70042. doi: 10.1002/bimj.70042 (PMC11898607; doi:10.1002/bimj.70042)
Supplement: Supplementary file 1 — Supporting Information [file BIMJ-67-e70042-s001.zip › density-based-ensemble-model-main/competitors_real_data/netlab-master/netlab-master/help/gsamp.htm]

Netlab Reference Manual gsamp


# gsamp

## Purpose

Sample from a Gaussian distribution.

## Synopsis

```
x = gsamp(mu, covar, nsamp)
```

## Description

`x = gsamp(mu, covar, nsamp)` generates a sample of size `nsamp`
from a `d`-dimensional Gaussian distribution. The Gaussian density
has mean vector `mu` and covariance matrix `covar`, and the
matrix `x` has `nsamp` rows in which each row represents a
`d`-dimensional sample vector.

## See Also

`gauss`, `demgauss`

---

**Pages:**
Index


---

Copyright (c) Ian T Nabney (1996-9)
